# Safety, tolerability, and biomarkers of the treatment of mice with aerosolized Toll-like receptor ligands

**DOI:** 10.3389/fphar.2014.00008

**Published:** 2014-02-06

**Authors:** Victoria Y. Alfaro, David L. Goldblatt, Gabriella R. Valverde, Mark F. Munsell, Lee J. Quinton, Adam K. Walker, Robert Dantzer, Atul Varadhachary, Brenton L. Scott, Scott E. Evans, Michael J. Tuvim, Burton F. Dickey

**Affiliations:** ^1^Department of Pulmonary Medicine, Unit 1462, The University of Texas MD Anderson Cancer Center, Houston, TXUSA; ^2^Department of Biostatistics, The University of Texas MD Anderson Cancer Center, Houston, TXUSA; ^3^The Pulmonary Center, Boston University School of Medicine, Boston, MAUSA; ^4^Department of Symptom Research, The University of Texas MD Anderson Cancer Center, Houston, TXUSA; ^5^AlphaDev, LLC, Houston, TXUSA; ^6^Pulmotect, Inc., Houston, TXUSA

**Keywords:** pneumonia, innate immunity, Toll-like receptor, oligodeoxynucleotide, lipopeptide, aerosol, myeloablation

## Abstract

We have previously discovered a synergistically therapeutic combination of two Toll-like receptor ligands, an oligodeoxynucleotide (ODN) and Pam2CSK4. Aerosolization of these ligands stimulates innate immunity within the lungs to prevent pneumonia from bacterial and viral pathogens. Here we examined the safety and tolerability of this treatment in mice, and characterized the expression of biomarkers of innate immune activation. We found that neutrophils appeared in lung lavage fluid 4 h after treatment, reached a peak at 48 h, and resolved by 7 days. The peak of neutrophil influx was accompanied by a small increase in lung permeability. Despite the abundance of neutrophils in lung lavage fluid, only rare neutrophils were visible histopathologically in the interstitium surrounding bronchi and veins and none were visible in alveolar airspaces. The cytokines interleukin 6 (IL-6), tumour necrosis factor, and Chemokine (C-X-C motif) ligand 2 rose several hundred-fold in lung lavage fluid 4 h after treatment in a dose-dependent and synergistic manner, providing useful biomarkers of lung activation. IL-6 rose fivefold in serum with delayed kinetics compared to its rise in lavage fluid, and might serve as a systemic biomarker of immune activation of the lungs. The dose–response relationship of lavage fluid cytokines was preserved in mice that underwent myeloablative treatment with cytosine arabinoside to model the treatment of hematologic malignancy. There were no overt signs of distress in mice treated with ODN/Pam2CSK4 in doses up to eightfold the therapeutic dose, and no changes in temperature, respiratory rate, or behavioral signs of sickness including sugar water preference, food disappearance, cage exploration or social interaction, though there was a small degree of transient weight loss. We conclude that treatment with aerosolized ODN/Pam2CSK4 is well tolerated in mice, and that innate immune activation of the lungs can be monitored by the measurement of inflammatory cytokines in lung lavage fluid and serum.

## INTRODUCTION

Infectious pneumonia exacts a high burden of morbidity and mortality worldwide (Mizgerd, 2008; Lozano et al., 2012). Efforts to protect populations from pneumonia have focused historically on antibiotics and vaccine-enhanced adaptive immunity. Using aerosolized bacterial lysates, we found that innate defenses of the lungs of mice can be stimulated therapeutically to induce a high level of resistance to microbial infection (Clement et al., 2008; Tuvim et al., 2009; Evans et al., 2010a,b). Resistance to infection rose rapidly after stimulation, reached a maximum by 4 h, remained at a high level for several days, and then slowly declined to baseline after several more days. Resistance to infection was accompanied by transient inflammation within the lungs, including an influx of neutrophils and cytokines into lavage fluid (Clement et al., 2008; Tuvim et al., 2009). However treatment appeared to be well tolerated with no apparent adverse behavioral effects, only a minimal rise of cytokines in serum, and resolution of lung inflammation within a week (Clement et al., 2008; Tuvim et al., 2009).

Subsequently, we found that genetic deletion of the innate immune adaptor MyD88 resulted in a complete loss of inducible resistance in response to aerosolized bacterial lysates (Duggan et al., 2011). This suggested that a subset of Toll-like receptor (TLR) ligands played a dominant role in the induction of resistance. We screened synthetic TLR ligands and found that even though no single aerosolized ligand was capable of inducing a high level of resistance, a combination of two particular ligands induced a high level of resistance to bacterial and viral infection of the lungs (Duggan et al., 2011; Tuvim et al., 2012; Cleaver et al., 2014). This combination consisted of a Class C oligodeoxynucleotide (ODN), which is a ligand for the TLR9 homodimer, and Pam2CSK4, which is a ligand for the TLR2/6 heterodimer. The concentration and ratio of the two components were then systematically varied to identify an optimal therapeutic formulation (Evans et al., 2011), which was found to be 1 μM ODN and 4 μM Pam2CSK4 (together designated O/P) in a nebulized solution of 4 ml. This aerosol therapy is being considered for clinical studies of its safety and efficacy in protecting immunocompromised subjects against opportunistic lung infections and normal subjects against virulent emerging and bioterror pathogens. Here we report the results of studies in mice to determine the safety and tolerability of aerosolized O/P and to identify biomarkers of lung innate immune activation that could help guide dosing in clinical trials.

## MATERIALS AND METHODS

### ANIMALS AND REAGENTS

All mice were handled in accordance with the policies of the Institutional Animal Care and Use Committee of The University of Texas MD Anderson Cancer Center. Adult (5–8 weeks old) male and female Swiss Webster (SW) and C57BL/6 mice (all from Charles River) were used. Mice were housed in plastic cages up to 5 per cage with ¼ inch Anderson Bed-O-Cob bedding (Lab Supply) on a 12 h light/dark cycle (7 am to 7 pm) with access to Regular Purina Rodent Diet 5001 (Lab Supply) and water *ad libitum* except as noted for behavioral studies. Where applicable, mice were euthanized by exsanguination under deep anesthesia with 2,2,2-tribromoethanol (Avertin, 500 mg/kg body weight) injected intraperitoneally (i.p.). All reagents were obtained from Sigma-Aldrich, except as indicated.

### AEROSOL TREATMENTS

Oligodeoxynucleotide 5′ TCG TCG TCG TTC GAA CGA CGT TGA T 3′ as the sodium salt on a nuclease-resistant phosphorothioate backbone (ODN M362) was purchased from TriLink BioTechnologies, and 2,3-bis(palmitoyloxy)-2-propyl-Cys-Ser-Lys-Lys-Lys-Lys-OH (Pam2CSK4) as the trifluoroacetic acid salt was purchased from Bachem. To treat the animals, ODN and Pam2CSK4 in indicated amounts were separately dissolved in 3 mL of endotoxin-free sterile water and then combined and placed in an Aerotech II nebulizer (Biodex Medical Systems). The nebulizer was driven by 10 L/min of air supplemented with 5% CO_2_ to promote deep breathing, and was connected by polyethylene tubing (30 cm × 22 mm) to a 10 L polyethylene exposure chamber that was vented to a biosafety hood. Mice were exposed to the aerosols for 20 min, resulting in the nebulization of approximately 4 ml of O/P solution. Control mice (prior to treatment for kinetic experiments and no O/P for dose–response experiments) were exposed to aerosols of water alone for 20 min. For estimation of the amount of O/P deposited within the lungs of mice in the aerosol chamber, we used Guyton’s formula predicting deposition of 0.2% of a delivered aerosol (Bide et al., 2000), together with our own measurement of 0.05% using aerosolized solutions of Evans Blue, to estimate aerosol deposition at 0.1%.

### LUNG LAVAGE FLUID AND SERUM ANALYSES

Lung lavage fluid was obtained by instilling, collecting and combining two aliquots of 1 mL each of PBS through a 20 gauge Luer stub adapter cannula (BD Biosciences) inserted through rings of the exposed trachea of anesthetized animals at the indicated time points. Total leukocyte count was determined using a hemocytometer (Hausser Scientific), and differential count by cytocentrifugation (CytoSpin 4, Thermo Fischer Scientific) of 300 μL of lavage fluid at 300 × *g* for 5 min followed by Wright-Giemsa staining. For cytokine analysis, the remaining lavage fluid was centrifuged at 1000 × *g* for 10 min and the cell-free supernatant collected and frozen. For serum cytokine measurement, blood was obtained from anesthetized mice by cardiac puncture and allowed to coagulate, then centrifuged and frozen. Cytokine concentrations in lavage fluid and serum were measured in duplicate by multiplexed sandwich enzyme-linked immunosorbent assay (ELISA) using SearchLight Array Services (Aushon Biosystems).

### HISTOLOGIC ANALYSES

Lungs were inflated at 15 cm H_2_O pressure with 10% buffered formalin and fixed *in situ *for 5 min at room temperature, then removed from the thoracic cavity and fixed overnight at 4 °C. Fixed lungs were embedded in paraffin and cut into 5 μm sections and placed on glass slides. Sections were dewaxed and rehydrated, and tissues were stained with Harris’ hematoxylin and eosin (H&E) to examine cellular elements, Masson’s trichrome (MTC) stain or picrosirius red (PSR) to examine collagen, and periodic acid fluorescent Schiff’s reagent (PAFS) to examine mucin accumulation as described (Moghaddam et al., 2008).

### MYELOABLATION AND INFECTIOUS CHALLENGE

Mice were injected i.p. four times (days -8, -5, -3, -1) with cytosine arabinoside, 100 mg/kg body weight, as described (Clement et al., 2008). On day 0, mice were challenged with aerosolized *Pseudomonas aeruginosa* targeted to LD_80_–LD_100_ as described (Duggan et al., 2011). Mice were examined twice daily by veterinarians blinded to their treatments and euthanized if found distressed or removed from their cages if found dead. The numbers of surviving mice were counted daily by investigators.

### LUNG PERMEABILITY ASSESSMENT

Lung permeability was measured three ways – total protein in lung lavage fluid, the amount of systemically administered dye in lung lavage fluid, and total lung weight. The protein concentration of lavage fluid was measured using a BCA Protein Assay Kit (Thermo Fischer Scientific) according to the manufacturer’s instructions. To measure dye translocation, anesthetized mice were injected intravenously (i.v.) in the tail vein with 100 μl Evans Blue dye (30 mM) in PBS with 0.1% bovine serum albumin, and the amount of dye appearing in lavage fluid was measured with a spectrophotometer at 620 nm. For a positive control, mice were given 3.7 mg of oleic acid combined with the dye injection. Wet lung weight was measured using mice that did not undergo lung lavage.

### EXPRESSION OF SAA1 IN LIVER BY QUANTITATIVE PCR

Serum amyloid A1 (SAA1) mRNA expression in the livers of challenged mice was measured using quantitative reverse transcription-polymerase chain reaction (qRT-PCR). Excised livers were snap frozen in liquid nitrogen, homogenized in TRIzol reagent (Life Technologies) using a Bullet Blender (Next Advance), and total RNA was isolated as described in the TRIzol protocol. Total RNA was further purified using the RNeasy kit (Qiagen). qRT-PCR was performed using either the CFX96 Real-Time System (Bio-Rad) or the StepOnePlus Real-Time PCR System (Applied Biosystem) in conjunction with the TaqMan RNA-to-C_T_ 1-step kit. Primer and probe sequences were the same as those used previously (Quinton et al., 2009), and were purchased from Integrated DNA Technologies. Results were expressed as fold induction normalized to the content of 18s rRNA as described (Jones et al., 2006).

### PHYSIOLOGIC AND BEHAVIORAL ASSESSMENTS

Mice were weighed at the indicated time points after challenge using a laboratory scale, temperature was taken with a rectal thermometer (Sper Scientific, Cat # 800060), and respiratory rate was measured using a plethysmograph (Scireq) with mice monitored in the chamber for 2 min. Sickness in mice can be measured by decreases in sucrose preference, food intake, locomotor activity, and social exploration (O’Connor et al., 2009). A sucrose anhedonia test was conducted as described (Willner et al., 1992). Briefly, five male SW mice were housed in each cage in the presence of two water bottles, one containing only water and the other containing sucrose (1% w/v) in water. The placement of the bottles was alternated daily. Mice were acclimated for 1 week prior to an experiment lasting 4 days. Sucrose preference was calculated by dividing the amount of sucrose solution consumed by the total liquid consumed. *Escherichia coli* endotoxin was used as a positive control (50 μg/mouse i.p.). Food consumption, locomotor activity and social exploration were also measured as behavioral indices of distress as described (Kent et al., 1992; Mormede et al., 2004; O’Connor et al., 2009). For these studies mice were housed singly in standard shoebox cages with Harlan/Teklad Laboratory Grade Sani-Chips bedding in a temperature (23°C) and humidity (45–55%) controlled environment with a 12/12-h modified dark–light cycle (lights on at 10:00 pm). Aerosol treatments and i.p. injections were given at the beginning of the dark period at 10:00 am. Behavior was assessed during the dark cycle in the presence of a darkroom red light by observers blinded to the mouse treatments. The mice were fed irradiated Prolab Isopro RMH 3000 (LabDiet). Food and water were freely available and mice were handled for 1 week prior to experimentation. For a positive control of all the behavioral indices besides sucrose preference, mice were injected with a lower dose of *E. coli* endotoxin (20 μg/mouse i.p.). Food consumption was measured by weighing remaining food pellets at intervals during a 24 h period. Locomotor activity was assessed by placing mice into a new cage similar to the home cage but devoid of bedding. The cage was divided into four virtual quadrants, and the number of line crossings (“quadrant entries”) and rearing on hind legs (“rears”) were counted over a 5-min period. Social interaction was assessed immediately after the 6 and 24 h locomotor activity assessments by placing into the cage a novel mouse of the same sex and age that had not been treated with an aerosol or i.p. injection. Time spent interacting was scored over a 5-min period when the resident mouse initiated nose-to-nose interaction, anogenital sniffing, or climbing over or under the novel mouse.

### STATISTICAL METHODS

In all studies except those of histopathology, descriptive statistics were used to summarize the percentage change of mean values from controls. In all figures, error bars represent the standard error of the mean. For studies of lung lavage fluid and serum cytokine and cellular responses and hepatic SAA1 transcript responses, unpaired *t*-test was used to assess statistically significant changes from baseline with a two-sided significance level of 0.05. For analysis of synergistic interactions between ODN and Pam2CSK in the induction of lung lavage fluid cytokine responses, a two-factor ANOVA model was used with an interaction term. For mouse survival studies, Fisher’s exact test was used to assess statistically significant changes from untreated controls with a two-sided significance level of 0.05. For mouse weight and temperature studies, paired *t*-test was used to assess statistically significant changes from vehicle treated controls with a two-sided significance level of 0.05 at each time point; mixed effects regression methods were used to model the percentage change from baseline as a function of dose, study day, and the interaction between dose and study day. Food consumption, locomotor activity and social exploration were assessed using one-way repeated measures of ANOVA. *Post hoc* analyses were conducted using Fisher’s protected least significant difference. For all studies, animals were randomly assigned to treatment groups, and investigators conducting assessments of mice or harvested samples were blinded to treatments. All figures show a representative experiment that was performed at least two times except for measurement of lung lavage fluid cytokine responses in myeloablated mice, which was only performed once because of the distress to mice treated with cytotoxic chemotherapy.

## RESULTS

### KINETICS OF LUNG INFLAMMATION AFTER A SINGLE O/P EXPOSURE

**FIGURE 1:**
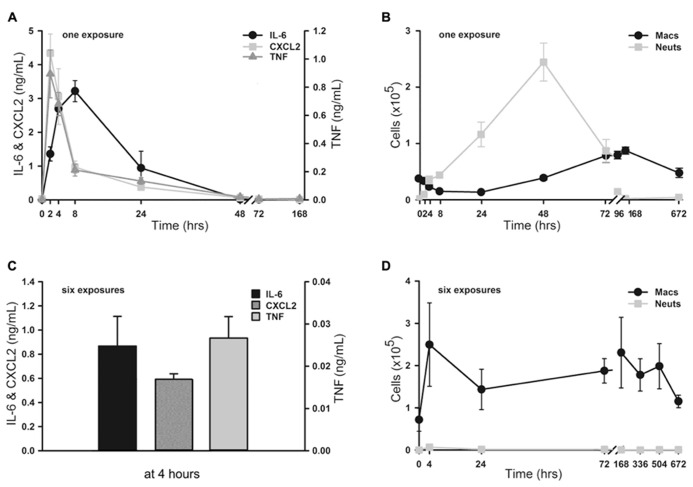
**Time-course of lung inflammatory responses to aerosolized O/P.** **(A,B)** Mice were exposed to a single aerosolized 1× dose of O/P, then sacrificed at the indicated times, and cytokine concentrations were measured in the supernatants **(A)** and macrophage and neutrophil numbers were measured in the cell pellets **(B)** of lung lavage fluid. Cytokine levels differed from baseline for IL-6 at 2, 4, and 8 h, for CXCL2 at 2, 4, 8, 24 h, and for TNF at 2, 4, 24, 48 h **(A)**. Leukocyte levels differed from baseline for macrophage at 96, 168, 672 h, and for neutrophils at 4, 8, 24, 48, 72 h **(B)**. **(C,D)** Mice were exposed twice weekly for 3 weeks to aerosolized 1× doses of O/P, then sacrificed at the indicated times after the last dose, and cytokine concentrations were measured in the supernatants **(C)** and macrophage and neutrophil numbers were measured in the cell pellets **(D)** of lung lavage fluid. All three cytokines levels differed from baseline at 4 h **(C)**. Macrophage levels differed from baseline only at 72 h, and neutrophil levels did not differ from baseline at any time **(D)**. (*n* = 3–4 for all experiments).

To assess the onset and duration of lung inflammation resulting from a single exposure to 4 ml of aerosolized 1 μM ODN and 4 μM Pam2CSK4 (hereafter, “1×” O/P), we measured cytokines and leukocytes in lung lavage fluid. Tumour necrosis factor (TNF) and chemokine (C-X-C motif) ligand 2 (CXCL2) increased 234-fold and 286-fold from baseline, respectively, with both cytokines peaking 2 h after treatment (**Figure 1A**). IL-6 peaked later, at 8 h, and increased 285-fold. Concentrations of all three cytokines returned to their low baseline levels 48 h after treatment. Neutrophil levels rose more slowly than cytokine levels, with no significant increase measureable at 2 h (**Figure 1B**). However there was a marked influx of neutrophils at 4 h, which peaked at 48 h, then declined at 72 h, with complete resolution at 7 days (168 h). In contrast, macrophages initially declined with a nadir at 24 h, then increased to a peak at 4–7 days (**Figure 1B**). Eosinophils were not observed at any time, but small numbers of lymphocytes (generally < 3% of total leukocytes) were observed from 2 to 7 days (not shown).

### KINETICS OF LUNG INFLAMMATION AFTER MULTIPLE O/P EXPOSURES

We then examined lung inflammation in response to repetitive exposure to aerosolized O/P given twice weekly for 3 weeks to mimic a prophylactic therapeutic regimen. The cytokine response 4 h after the last of six aerosol exposures (**Figure 1C**) was blunted as compared to the response 4 h after a single exposure (0.9 vs 2.7 ng/mL for IL-6, 0.6 vs 3.1 ng/mL for CXCL2, 0.03 vs 0.3 ng/mL for TNF). Remarkably, lung lavage fluid neutrophil levels (**Figure 1D**) barely rose at all at any time after six exposures. Lavage fluid macrophage levels rose 4 h after six exposures (**Figure 1D**), in contrast to the response to a single exposure (**Figure 1B**). They remained elevated for 3 weeks (504 h) before returning to baseline after 4 weeks (672 h).

### DOSE–RESPONSE RELATIONSHIPS BETWEEN LUNG INFLAMMATION AND O/P EXPOSURE

**FIGURE 2:**
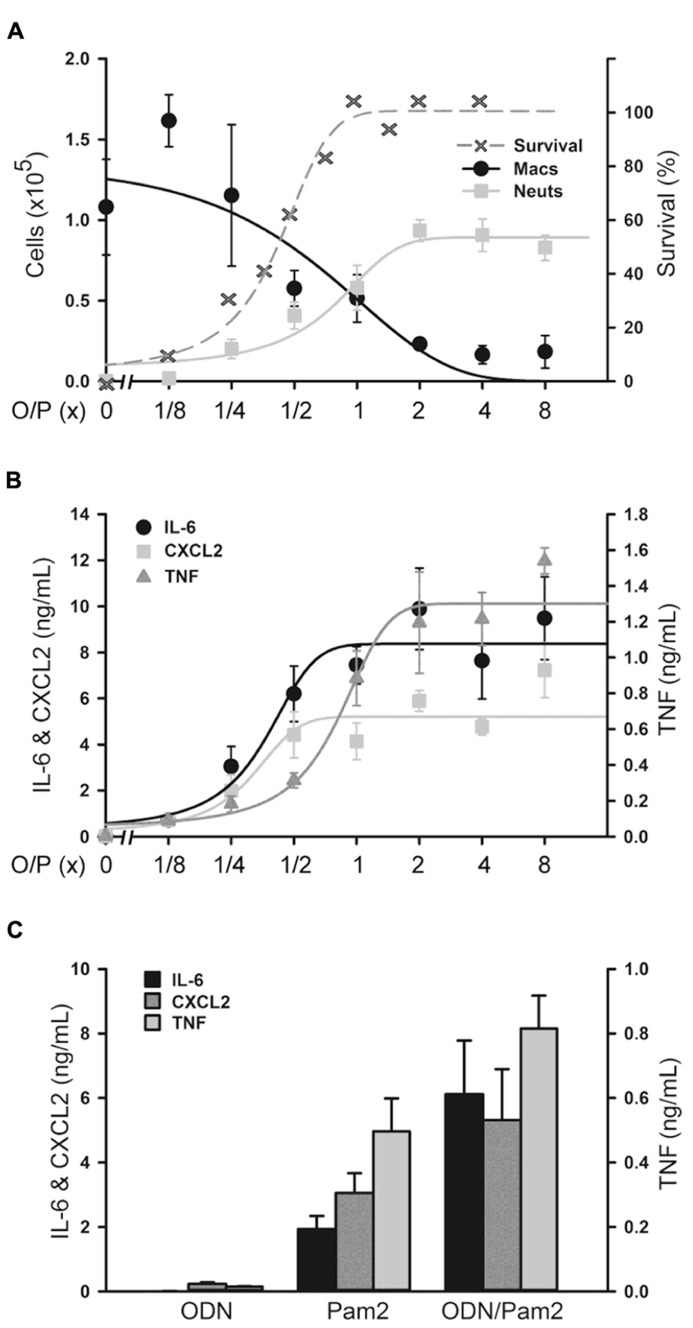
**Dose–response of lung inflammation to aerosolized O/P.** **(A,B)** Mice were exposed to increasing concentrations of O/P as indicated, then sacrificed after 24 h to measure macrophage and neutrophil numbers **(A)** or after 4 h to measure cytokine levels **(B)**. For comparison, the survival of mice exposed to increasing concentrations of O/P from challenge 24 h later with aerosolized *P. aeruginosa* is illustrated **(A)**. Neutrophil levels differed from baseline at concentrations ≥1/4×, survival levels at concentrations ≥1/2×, macrophage levels at concentrations ≥1×, and levels of all three cytokines at all concentrations. **(C)** Mice were exposed to aerosolized 1× ODN alone, Pam2 alone, or ODN and Pam2 together, and cytokine levels in lung lavage fluid were measured 4 h later. Levels of all cytokines differed from baseline in response to all treatments except IL-6 in response to ODN alone, and the levels of IL-6 (*p* = 0.012) and TNF (*p* = 0.025) but not CXCL2 (*p* = 0.185) in response to ODN and Pam2 together were more than additive for responses to ODN alone and Pam2 alone. (*n* = 3–4 for all experiments).

We next determined the dose–response relationship between O/P exposure and lung inflammatory responses across a 64-fold range of doubling concentrations (1/8×–8×) centered around the therapeutic 1× concentration. The highest concentration was 8× because a precipitate formed at higher concentrations. Lung lavage fluid neutrophil levels 24 h after O/P exposure showed a positively correlated sigmoidal dose–response curve, but macrophages showed a reciprocal response (**Figure 2A**). The plateau levels of the neutrophil and macrophage responses to the higher concentrations of O/P were reached by 2× O/P, and for comparison a representative efficacy (mouse survival) dose–response curve is also illustrated (**Figure 2A**). Cytokine levels were measured 4 h after treatment, a time point intermediate between the peak of TNF and CXCL2 at 2 h and the peak of IL-6 at 8 h. All three cytokines demonstrated saturable, sigmoidal dose–response curves (**Figure 2B**). The EC_50_ values of these responses are listed in **Table 1**, together with the more clinically relevant EC_90_ values. Importantly, the upper inflection point (EC_90_) of the dose–response curve for each cytokine (**Figure 2B** and **Table 1**) fell within one doubling concentration of the 1× therapeutic dose (**Figure 2A**, **Table 1**, and Evans et al., 2011), indicating that the cytokine dose–response relationships correlate with the resistance dose–response relationship (Duggan et al., 2011; Evans et al., 2011). Since ODN and Pam2CSK4 interacted synergistically to induce resistance to infection (Duggan et al., 2011; Evans et al., 2011), we tested whether they also interact synergistically to induce cytokine expression. ODN alone induced only very low levels of cytokines whereas Pam2CSK4 induced robust levels of IL-6, CXCL2, and TNF (**Figure 2C**), similar to their respective abilities to elicit neutrophil influx into lung lavage fluid (Duggan et al., 2011). Together, they synergistically increased cytokine levels (**Figure 2C**).

**Table 1:** Dose–response relationships of O/P.

Response	EC_50_	EC_90_
**Normal mice**
Host survival	0.42	1.02
Lavage neutrophil rise	0.74	1.38
Lavage macrophage fall	0.67	1.72
IL-6	0.37	0.66
CXCL2	0.30	0.78
TNF	0.80	1.46
**Myeloablated mice**
IL-6	0.17	0.23
CXCL2	0.13	0.15
TNF	0.20	0.31

*Values for the concentration of O/P, expressed as a fraction of 1× O/P (1 μM ODN M362 and 4 μM Pam2CSK4), resulting in 50% (EC_50_) and 90% (EC_90_) maximal host responses.*

### LUNG HISTOPATHOLOGY AFTER O/P EXPOSURE

**FIGURE 3:**
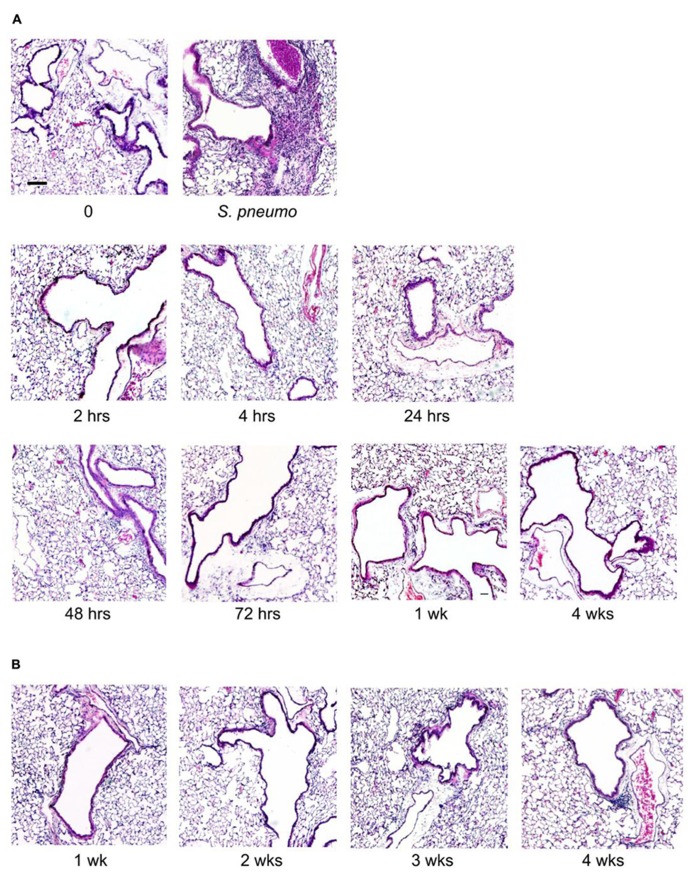
**Histopathology of mouse lungs in response to aerosolized O/P.** **(A)** Mice were exposed to a single aerosolized 1× dose of O/P, then sacrificed at the indicated times. Their lungs were fixed, embedded, and sectioned, then stained with H&E. For comparison, the lungs of a mouse sacrificed 24 h after challenge with aerosolized *S. pneumoniae* are illustrated. **(B)** Mice were exposed twice weekly for 3 weeks to aerosolized 1× doses of O/P, then sacrificed at the indicated times after the last dose, and their lungs were fixed, embedded, and sectioned, then stained with H&E. (*n* = 3–4 for all experiments).

Lung inflammation after aerosol O/P administration was also assessed histopathologically. Despite the influx of neutrophils into lavage fluid (**Figures 1B** and **2A**), no neutrophil infiltration into alveoli was perceptible on histologic analysis at any time point after a single exposure to O/P (**Figure 3A**). Small numbers of neutrophils were visible in the interstitial tissue of bronchovascular bundles from 4 to 72 h. In contrast, infection with *Streptococcus pneumoniae* as a positive control caused intense neutrophilic infiltration of interstitial tissue and alveoli at 24 h (**Figure 3A**), consistent with the more than 10-fold greater number of neutrophils in lung lavage fluid in this infection model than with O/P (not shown). The lungs of mice were also examined weekly for 4 weeks beginning 1 week after the last of six exposures to O/P given twice weekly for 3 weeks. No inflammatory cell infiltration of the lungs was apparent 1 week or later after repetitive O/P exposure (**Figure 3B**). Lung tissues were also examined for evidence of fibrosis using MTC and PSR stains, and fibrosis was not observed (not shown). Mucous metaplasia was not observed by PAFS staining or epithelial injury by H&E staining in single or multiple exposure experiments (not shown).

### ANTIMICROBIAL RESISTANCE AND BIOMARKERS IN MYELOABLATED MICE

**FIGURE 4:**
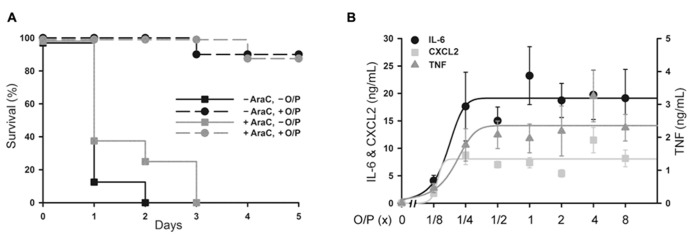
**Protection from *Pseudomonas *pneumonia and expression of biomarkers in myeloablated mice treated with aerosolized O/P.** **(A)** Mice were (black symbols) or were not (gray symbols) given four intraperitoneal injections of cytosine arabinoside (AraC) over 8 days to ablate neutrophils. Mice from both groups were then treated (circles) or not (squares) with a 1× dose of aerosolized O/P, then challenged 24 h later with aerosolized *P. aeruginosa*. Survival of mice treated with O/P differed from survival of untreated mice regardless of whether they received AraC (*n* = 10). **(B)** Mice myeloablated with cytosine arabinoside were exposed to increasing concentrations of O/P as indicated, then sacrificed after 4 h for the measurement of cytokine concentrations in lung lavage fluid. Levels of all cytokines differed from baseline in response to all treatments (*n* = 4).

Aerosolized O/P might be used to treat patients with acute leukemia undergoing induction of remission therapy because of their high susceptibility to pneumonia as a result of myelosuppression from both the leukemia and its treatment (Barreda et al., 2013). At a time when neutrophils are not available to participate in pathogen defense, we hypothesize that stimulation of the lung epithelium with aerosolized O/P could provide transient resistance to infection. To test this hypothesis, we used a mouse model of myeloablation with the antimetabolite cytosine arabinoside that is commonly used to treat hematologic malignancies. In this mouse model, no neutrophils are measureable in lung lavage fluid after exposure to an aerosolized bacterial lysate (Clement et al., 2008). Most mice treated with a single 1× dose of aerosolized O/P survived an otherwise lethal challenge with *P. aeruginosa* 24 h later, with no apparent difference in protection between control mice and those treated with cytosine arabinoside (**Figure 4A**). Aerosolized O/P also induced a dose-dependent increase in lung lavage cytokines in these mice (**Figure 4B**), similar to that observed in mice not treated with cytosine arabinoside (**Figure 2B**). Together, these results suggest that lung epithelial antimicrobial and inflammatory responses are preserved despite treatment with myeloablative chemotherapy.

### LUNG PERMEABILITY AFTER O/P EXPOSURE

**FIGURE 5:**
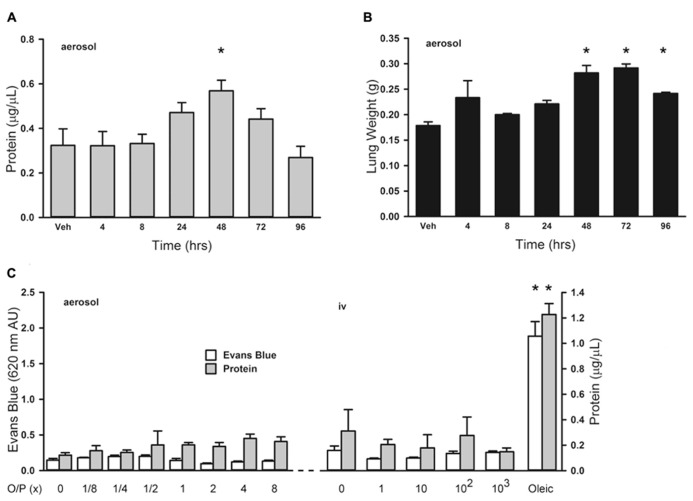
**Lung permeability changes in response to aerosolized O/P.** **(A,B)** Mice were exposed to a single 1× dose of aerosolized O/P, then sacrificed at the indicated times to measure protein concentration in lung lavage fluid **(A)** or lung weight **(B)**. Asterisks indicate differences in comparison to mice treated with vehicle alone (*n* = 4 in **A**, 3 in **B**). **(C)** Mice on the left were exposed to increasing concentrations of aerosolized O/P as indicated, and those on the right were injected intravenously with amounts of O/P estimated to be 1, 10, 100, or 1,000-fold the amount deposited in the lungs after a single aerosolized 1× dose. All mice were injected intravenously with Evans blue dye at the time of exposure to aerosolized or intravenous O/P, then sacrificed after 48 h for measurement of the concentration of protein and Evans blue dye in lung lavage fluid. For comparison, mice were injected with oleic acid intravenously to induce a substantial increase in lung permeability. Asterisks indicate differences in comparison to mice treated with vehicle alone (*n* = 4).

Treatment of leukemia can cause an increase in lung permeability leading to impaired gas exchange as a result of inflammation from tumor cell lysis and direct drug-induced lung injury (Tryka et al., 1982; Briasoulis and Pavlidis, 2001). We evaluated the effects of aerosolized O/P treatment on lung permeability to help determine whether it might aggravate the increase in lung permeability caused by tumor treatment. Initially we examined the time course of changes in lung permeability by measuring protein concentration in lung lavage fluid, and found a small transient increase 48 h after exposure to 1× aerosolized O/P (**Figure 5A**; 0 at 4 h, 3% at 8 h, 46% at 24 h, 75% at 48 h, 36% at 72 h, -17% at 96 h). This increase in lung permeability was also observed by measuring wet lung weight (**Figure 5B**). Next we examined the dose–response relationship 48 h after O/P aerosolization by measurement of both Evans Blue extravasation and protein concentration in lung lavage fluid (**Figure 5C**, left side). There appeared to be a small increase in protein concentration (40% for 1× and 60% for 8× O/P) as was observed in the kinetic experiment, but it was not statistically significant due to the multiple comparisons. There was no increase in Evans Blue extravasation, and no apparent respiratory distress in mice. For comparison, we injected oleic acid i.v. (**Figure 5C**, right side), which is a well-established model of increased permeability due to lung injury (Wang et al., 2008). Oleic acid caused much greater increases in Evans Blue extravasation (850%) and protein concentration (392%) in lung lavage fluid than any dose of aerosolized O/P, and caused obvious respiratory distress. We also injected O/P i.v. to assess whether the permeability changes induced by aerosolized O/P are due to topical inflammation within the lungs or to systemic inflammation from O/P that might translocate into the circulation. Injection of up to 10^3^-fold the amount of O/P we calculate is deposited in the lungs by a 1× aerosol (1 μm ODN and 4 μm Pam2 × 4 ml × 0.1% deposition = 4 pmol ODN and 16 pmol Pam2CSK4) did not result in a measureable increase in Evans Blue extravasation or protein concentration or in apparent respiratory distress (**Figure 8C**). Together, these results suggest that aerosolized O/P induces a mild increase in lung permeability due to topical rather than systemic inflammation.

### SYSTEMIC CYTOKINES AFTER O/P EXPOSURE

**FIGURE 6:**
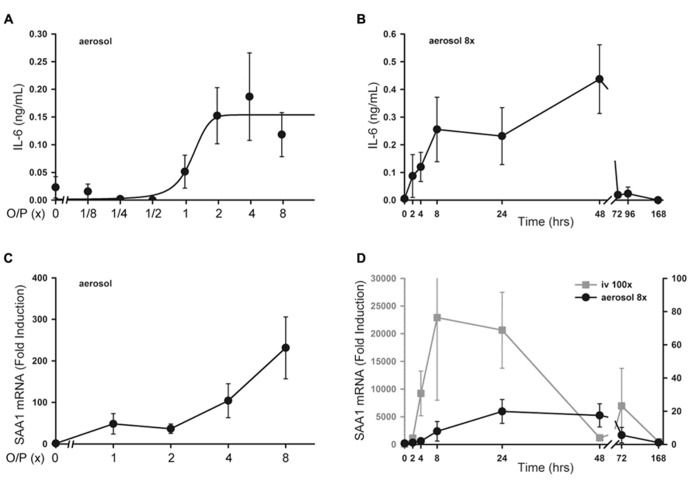
**Systemic inflammatory responses to aerosolized O/P.** **(A)** Mice were exposed to increasing concentrations of O/P as indicated, then sacrificed after 4 h to measure cytokine levels in serum. IL-6 levels differed from baseline at concentrations of O/P ≥2×. **(B)** Mice were exposed to a single aerosolized 8× dose of O/P, then sacrificed at the indicated times, and the IL-6 concentration in serum was measured. The level of IL-6 differed significantly from baseline only at 48 h. **(C)** Mice were exposed to increasing concentrations of O/P as indicated, then sacrificed after 4 h to measure SAA1 transcript levels in their livers. Levels of SAA1 transcripts differed from baseline at concentrations of O/P ≥2×. **(D)** Mice were exposed to a single aerosolized 8× dose of O/P (black circles, Y-axis on the right), then sacrificed at the indicated times to measure SAA1 transcript levels in their livers. For comparison, mice were injected intravenously (gray squares, Y-axis on the left) with an amount of O/P estimated to be 100-fold the amount deposited in the lungs after a single aerosolized 8× dose. Levels of SAA1 transcripts differed from baseline at 24 and 48 h. (*n* = 4–5 for all experiments).

There was no significant increase in serum TNF or CXCL2 levels above baseline measured 4 h after exposure to aerosolized O/P at any concentration up to 8× (not shown). Serum IL-6 rose fivefold after exposure to 2× O/P, but this level did not further increase with exposure to 4× or 8× O/P (**Figure 6A**). The kinetics of the rise in serum IL-6 were delayed in comparison to the rise in lavage fluid IL-6, with the peak serum level occurring at 48 h (**Figure 6B**) compared to the peak lavage fluid level at 8 h (**Figure 1A**). The low levels of serum cytokines in response to aerosolized O/P are similar to what we previously observed with aerosolized bacterial lysates (Clement et al., 2008; Tuvim et al., 2009), confirming that inflammation resulting from topical activation of innate immunity within the lungs remains mostly confined within the lungs. Nonetheless, IL-6 might serve as a systemic biomarker of lung epithelial activation.

### HEPATIC SAA1 TRANSCRIPTS AFTER O/P EXPOSURE

As a sensitive index of systemic inflammation, we measured the expression of transcripts of the acute phase reactant SAA1 in liver (Quinton et al., 2009). Following the delivery of increasing amounts of O/P, there was a small dose-dependent increase in SAA1 mRNA expression in livers taken 4 h after the aerosolization (**Figure 6C**). Similar to the kinetics of the rise in serum IL-6, the peak level of SAA1 transcripts in the liver occurred between 24 and 48 h (**Figure 6D**, gray line). To assess whether the hepatic expression of SAA1 was due to translocation of aerosolized O/P into the systemic circulation or to systemic transmission of the local lung inflammatory response, we injected into the tail vein the amount of O/P (4 pmol ODN and 16 pmol Pam2CSK4) we estimated is deposited within the lungs of a mouse after aerosolization of 1× O/P. This did not result in a measurable increase in SAA1 transcripts in the liver (not shown). As a positive control, we injected 100-fold the amount of O/P deposited by a 1× aerosol, which induced a higher and more rapid rise in liver SAA1 transcript levels than aerosolization of 8× O/P (**Figure 6D**, black line). Mice did not show behavioral signs of distress in response to the tail vein injection of 100× O/P. Together, these results suggest that the mild hepatic SAA1 response to aerosolized O/P is due to systemic transmission of the local lung inflammatory reaction rather than to translocation of O/P into the circulation, and that this mild systemic inflammatory response is subclinical.

### ANIMAL PHYSIOLOGY AFTER O/P EXPOSURE

**FIGURE 7:**
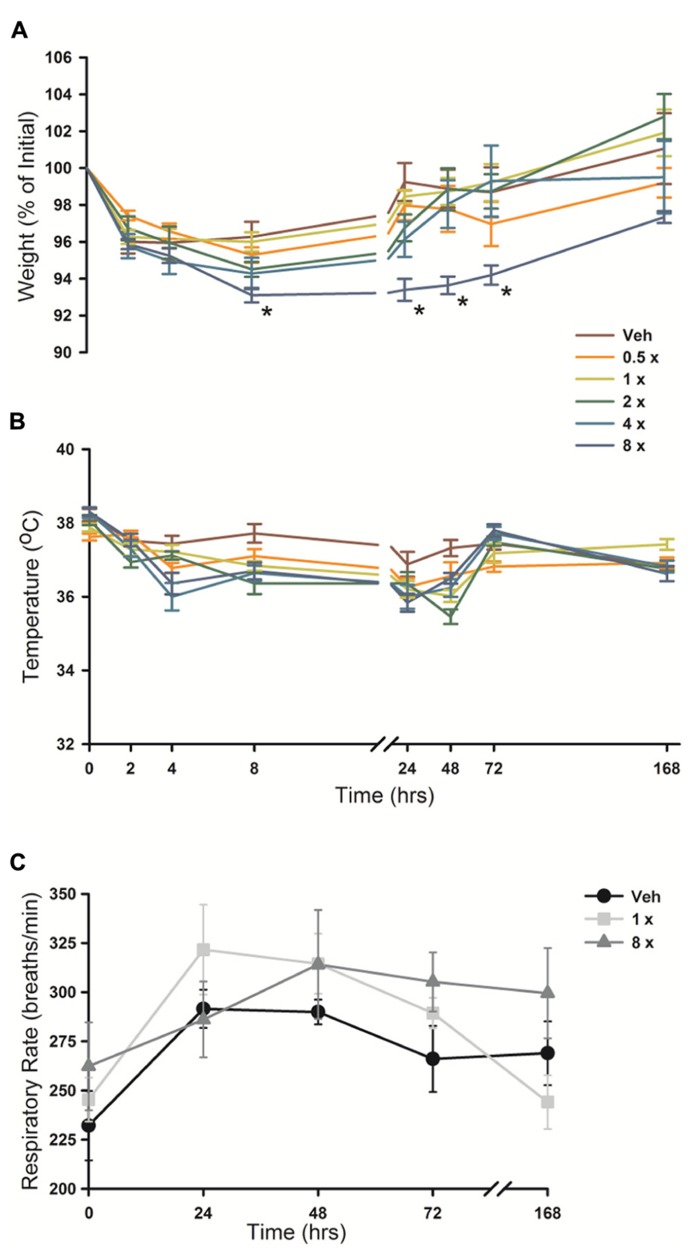
**Physiologic responses to aerosolized O/P.** **(A,B)** The weight **(A)** and temperature **(B)** of mice exposed to increasing concentrations of O/P as indicated by the various colored lines was recorded at the indicated times. Asterisks indicate differences in comparison to mice treated with vehicle alone. **(C)** The respiratory rate of mice treated with aerosolized vehicle alone (water, black circles), or 1× (gray squares) or 8× (gray triangles) O/P. There were no differences in comparison to mice treated with vehicle alone. (*n* = 4–5 for all experiments).

Animal weight, temperature and respiratory rate were measured following O/P administration. There was a small (2–5%) decrease in weight in all mice from 2 to 24 h after aerosol exposure (**Figure 7A**), including those treated only with aerosolized water (vehicle), that we attributed to the stress of handling and the fact that mice huddle in a group and do not drink or eat during nebulization. No O/P aerosol treatment group showed a significant decrease in weight compared to the group treated with aerosolized water except the 8× O/P aerosol group that showed a 7% decrease. There was a similar small decrease in temperature in all aerosol treatment groups, including mice treated with water alone, but there were no significant further decreases in any group treated with aerosolized O/P as compared to mice treated with water alone (**Figure 7B**). There were no significant differences in respiratory rate for mice treated with therapeutic (1×) or high (8×) concentrations of aerosolized O/P as compared to mice treated with aerosolized water (**Figure 7C**). Together these results suggest that aerosolized O/P causes only minimal changes in physiology.

### ANIMAL BEHAVIOR AFTER O/P EXPOSURE

Since mouse behavioral models can provide sensitive indicators of inflammation or distress, we evaluated the impact of aerosolized O/P using multiple well-established models. There was no loss of preference for lightly sweetened water (1% sucrose) over unsweetened water in mice treated with any concentration of aerosolized O/P in comparison to mice treated with aerosolized water (**Figure 6A**). As a positive control, mice that received 50 μg of endotoxin by i.p. injection almost entirely lost their preference for sweetened water during the first 24 h after injection (**Figure 6A**). There was an approximately 30% decrease in total water consumption in all groups of mice during the first 24 h after aerosol exposure with no difference between those exposed to vehicle alone compared to those exposed to O/P (not shown). Decreased food consumption, social interaction, and locomotion (quadrant entry and rears) are additional indicators of distress in mice. Neither 1× nor 8× aerosolized O/P caused any decrease in food consumption (**Figure 6B**), social interaction (**Figure 6C**), or locomotion (**Figures 6D,E**) as compared to aerosolized water, though 20 μg of i.p. endotoxin caused a significant decrease in each of these measures at early time points. Together, these results suggest that aerosolized O/P is well tolerated by mice.

## DISCUSSION

We have previously identified a combination of TLR ligands that effectively induces resistance to microbial infection (Duggan et al., 2011; Evans et al., 2011; Tuvim et al., 2012; Cleaver et al., 2014). While it is formally possible to separate host responses that directly mediate pathogen killing (e.g., expression of antimicrobial peptides and generation of reactive oxygen species) from inflammatory responses that recruit leukocytes (e.g., expression of chemokines and generation of eicosanoids), the stimulation of innate immunity generally induces resistance and inflammation together (Evans et al., 2010b). Therefore, pragmatically at this point in time, therapeutic stimulation of innate immunity to treat infection depends upon whether accompanying inflammatory responses are acceptable. Here we have analyzed in mice, the species in which we have done most of our work on inducible resistance, the safety and tolerability of treatment with O/P.

## LUNG INFLAMMATION

The lungs are the site of delivery of aerosolized O/P, so should be the site of most intense inflammation. At therapeutic doses, O/P caused an acute inflammatory response characterized by a rise in inflammatory cytokines and neutrophils in lung lavage fluid, but with minimal tissue infiltration by leukocytes histopathologically (**Figure 1**). The cytokines in lavage fluid returned to baseline within 2 days and the neutrophils within 4 days. The presence of lavage fluid neutrophils was paralleled by a small transient increase in lung permeability (**Figure 5**), which did not result in respiratory distress or a rise in respiratory rate (**Figure 7C**). The transient rise in lavage fluid neutrophils contrasts with an initial fall in macrophages, possibly due to their mobilization to local lymph nodes or increased adhesion to alveolar walls (Geissmann et al., 2010), followed by a late rise in macrophages that is probably part of the process of clearance of inflammation (**Figure 1B**).

The moderate intensity of lung inflammation seen here could be expected from the mild inflammation we observed with aerosolization of a TLR9 agonist alone (Duggan et al., 2011) and others observed with intratracheal instillation of TLR9 agonists (Schwartz et al., 1997; Duechs et al., 2011) in mice, combined with the moderate inflammation we observed with aerosolization of a TLR2 agonist alone (Duggan et al., 2011) and others observed with intratracheal instillation of a TLR2 agonist (Schwartz et al., 1997; Duechs et al., 2011). However these findings stand in contrast to the severe lung inflammation reported with intranasal administration to mice of a TLR9 agonist (Campbell et al., 2009). The agonist used in that study was a Class B ODN with higher activity toward rodents than primates (Campbell et al., 2009), whereas we used a Class C ODN with similar activity toward rodents and primates, but Duechs et al. (2011) also used a Class B ODN with higher activity toward rodents than primates without causing severe inflammation. Another possible explanation of the discrepancy is the higher dose used by Campbell et al. (2009; 5 mg/kg, yielding 150 μg for a 30 *g* mouse, and estimated lung deposition of 50% for intranasal instillation = 75 μg), compared to the low dose in our study (8.58 μg/ml in nebulizer × 4 ml × 0.1% deposition = 34 ng). However, the dose used by Duechs et al. (2011) (1 mg/kg, yielding 30 μg for a 30 *g* mouse, and estimated lung deposition of 100% for intratracheal instillation = 30 μg) was only slightly lower than the dose used by Campbell et al. (2009) yet caused only mild inflammation. Thus, the cause of the discrepancy between the severe lung inflammation in response to a TLR9 agonist reported by Campbell et al. (2009) and the mild inflammation reported by us and others is not apparent.

An interesting feature of lung inflammation in response to aerosolized O/P was its self-limited nature. The rise in lavage cytokines and neutrophils was dose-dependent up to the therapeutic dose, but plateaued at higher doses (**Figure 2**). This suggests that TLR2/6 and TLR9 receptors in the airways are saturated at this dose of ligands, or that downstream signaling pathways leading to both antimicrobial and cytokine responses are maximally activated. Even more striking was the degree of tachyphylaxis with repetitive dosing, such that lavage neutrophils barely rose at all after six doses and cytokines rose only a fraction as high as after a single dose (**Figures 1C,D**). These findings are consistent with our earlier finding of tachyphylaxis of lung inflammation (Moghaddam et al., 2008), though not of antimicrobial resistance (Tuvim et al., 2009; Evans et al., 2010a), induced by repetitive exposure to an aerosolized bacterial lysate. Others have similarly found tachyphylaxis of inflammation but not of antimicrobial responses in response to a TLR agonist *in vitro* (Foster et al., 2007). Together, these findings indicate that lung inflammation from aerosolized O/P is limited in severity, in duration, and in response to repetitive exposure.

## SYSTEMIC RESPONSES

**FIGURE 8:**
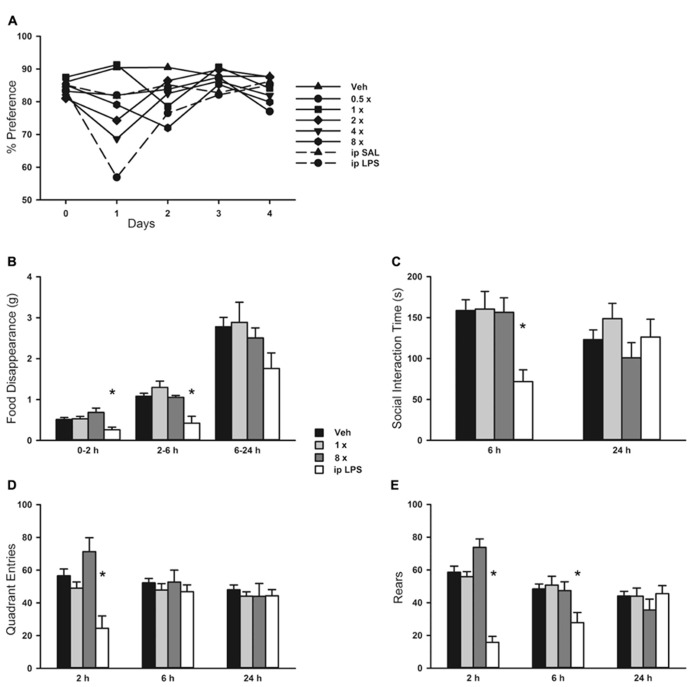
**Behavioral response to aerosolized O/P.** **(A)** Mice were exposed to increasing concentrations of aerosolized O/P (symbols with solid lines) or to saline solution (SAL) or lipopolysaccharide (LPS) given by intraperitoneal (i.p.) injection (symbols with dashed lines). For the next 4 days, their preference for lightly sweetened water (1%) over unsweetened water was measured. Statistical analysis was not performed because data points represent measurements from a single cage (*n* = 5). **(B–E)** Mice were exposed to 1× or 8× doses of aerosolized O/P and compared to mice exposed to aerosolized vehicle (water) or injected i.p. with LPS. The rate of food disappearance **(B)**, seconds per 5 min spent in social interaction with a novel untreated age and sex matched mouse introduced into the cage **(C)**, and the number of quadrant entries **(D)** and rears **(E)** per 5 min of mice introduced into novel cages were measured for the next 24 h. Asterisks indicate differences in comparison to mice treated with vehicle alone (*n* = 8).

There was a small rise in serum IL-6 after aerosolized O/P (**Figures 6A,B**), but no detectable rise in TNF or CXCL2, suggesting that inflammation is mostly contained within the lungs. This is similar to our prior finding of a minimal rise in serum cytokines after exposure to an aerosolized bacterial lysate (Tuvim et al., 2009), and consistent with the lack of systemic antimicrobial resistance after stimulation of lung innate immunity (Clement et al., 2008). Even the expression of SAA1 transcripts in the liver, a highly sensitive indicator of systemic inflammation (Quinton et al., 2009) and the most highly upregulated gene in the lungs of mice treated with O/P (Evans et al., 2010a), rose only minimally after aerosolized O/P (**Figures 6C,D**). This small degree of systemic inflammation is more likely due to the relay of local inflammatory responses from the lungs rather than translocation of aerosolized O/P into the systemic circulation because intravenous injection of quantities of O/P many times greater than those calculated to be deposited in the lungs after aerosolization resulted in only modest rises in hepatic SAA1 transcripts (**Figure 6D**) and lung permeability (**Figure 5C**). The mild systemic inflammation after aerosolized O/P was not reflected in changes in the respiratory rate or core temperature of mice (**Figures 7B,C**), though there was a small transient reduction in weight (**Figure 7A**) and water consumption (not shown) in mice treated with an 8× dose. Behavioral models of stress showed no differences between mice treated with vehicle and those treated with aerosolized O/P, even though those models were sensitive to low doses of intravenous lipopolysaccharide (LPS; **Figure 8**). These findings of minimal systemic inflammation stand in contrast to the severe macrophage activation syndrome with cytokine storm, lymphoid follicle destruction, splenomegaly and hepatitis that occurred after repetitive i.p. injection of 50 μg of a Class B ODN in mice (Heikenwalder et al., 2004; Behrens et al., 2011; Canna et al., 2013). It appears that the activation of innate immunity at mucosal surfaces differs fundamentally from its activation within the body in the intensity of systemic inflammation evoked since we find substantial inflammation within the lung lumen but minimal transmission systemically.

## BIOMARKERS

Pharmacodynamic measures of the activity of aerosolized O/P could be important in determining therapeutic dose levels in clinical trials. The close correlation between the rising portion of the dose–response curves for antimicrobial efficacy and lung lavage cytokines in mice (**Figure 2**) suggests that induced sputum or bronchoalveolar lavage fluid might be sampled in human subjects for this purpose. The synergistic increase in lavage fluid cytokine levels when ODN and Pam2CSK4 are administered together compared to their levels when each agonist is administered alone (**Figure 2C**) mirrors the synergistic induction of antimicrobial resistance and neutrophil recruitment by these ligands (Duggan et al., 2011; Tuvim et al., 2012; Cleaver et al., 2014), further validating the utility of these cytokines as biomarkers of pharmacodynamic activity. The preservation of resistance (**Figure 4A** and Duggan et al., 2011; Tuvim et al., 2012; Cleaver et al., 2014) and cytokine (**Figure 4B**) responses in myeloablated mice suggests that clinical studies in patients with hematologic malignancies undergoing myeloablative chemotherapy could also be guided by lung lining fluid cytokine biomarkers. The rise in serum IL-6 in mice (**Figure 6**) suggests that serum cytokine measurements in human subjects might also be useful. Whereas the dose–response relationship between antimicrobial efficacy and cytokine levels is close, some cytokines rise slightly faster than the induction of antimicrobial resistance [CXCL2 and TNF in **Figure 1A**, compared to the kinetics of resistance in Clement et al. (2008) **Figure 1**], and some rise slower (IL-6 in **Figure 1A**). More importantly, all three cytokines decline to baseline by 24–48 h while antimicrobial resistance remains high several days longer (Clement et al., 2008; Tuvim et al., 2009, 2012; Evans et al., 2010a; Duggan et al., 2011). Thus, cytokine measurement should be useful for dose finding but not for the assessing the kinetics of activity. Measurement of a key antimicrobial activity would serve as a better kinetic biomarker, but the essential effectors of inducible epithelial resistance are not yet known (Evans et al., 2010b, 2011; Cleaver et al., 2014).

## CONCLUSION

Exposure of mice to an aerosolized combination of TLR2/6 and TLR9 ligands induces transient and self-limited neutrophilic inflammation within the lungs that is associated with minimal systemic inflammation or physiologic and behavioral responses. The dose-dependent rise in cytokines in lung lining fluid and serum could serve as biomarkers of pharmacodynamic effects for dose-finding in clinical trials. Together, these findings suggest that it may be feasible to use aerosolized TLR ligands to treat immunocompromised subjects to prevent opportunistic lung infections or normal subjects to attenuate lung infections with virulent pathogens.

## Conflict of Interest Statement

Scott E. Evans, Michael J. Tuvim, and Burton F. Dickey are inventors of a technology to deliver aerosolized TLR ligands to induce resistance to microbial infection of the lungs; this technology has been licensed by MD Anderson Cancer Center to Pulmotect, Inc. (Houston, TX, USA), in which Scott E. Evans, Michael J. Tuvim, Burton F. Dickey, Atul Varadhachary, and Brenton L. Scott have ownership interests, and which has sponsored research in the laboratories of Scott E. Evans, Michael J. Tuvim, and Burton F. Dickey. Robert Dantzor consults for Ironwood Pharma (Cambridge, MA, USA). The other authors have no conflicts of interest.

## Abbreviations

i.p.: intraperitoneally
i.v.: intravenously
LPS: lipopolysaccharide
ODN: oligodeoxynucleotide
ODN M362: TCG TCG TCG TTC GAA CGA CGT TGA T
O/P: ODN M362 and Pam2CSK4 at a 1:4 molar ratio
Pam2CSK4: 2,3-bis(palmitoyloxy)-2-propyl-Cys-Ser-Lys-Lys-Lys-Lys-OH
qRT-PCR: quantitative reverse transcription-polymerase chain reaction
SAA1: serum amyloid A1
TLR: Toll-like receptor
